# Detection of Insomnia and Its Relationship with Cognitive Impairment, Depression, and Quality of Life in Older Community-Dwelling Mexicans

**DOI:** 10.3390/diagnostics13111889

**Published:** 2023-05-28

**Authors:** Elsa Correa-Muñoz, Raquel Retana-Ugalde, Víctor Manuel Mendoza-Núñez

**Affiliations:** 1 Unidad Investigación en Gerontología, Facultad de Estudios Superiores Zaragoza, Universidad Nacional Autónoma de México, Mexico City 09230, Mexico; 2 Facultad de Humanidades, Ciencias Sociales y Empresariales, Universidad Maimónides, Hidalgo 775—CABA, Buenos Aires B1686IGC, Argentina

**Keywords:** insomnia, cognitive impairment, depression, quality of life, sleep quality, older adult

## Abstract

Sleep disturbances are one of the most frequent health problems in old age, among which insomnia stands out. It is characterized by difficulty falling asleep, staying asleep, frequent awakenings, or waking up too early and not having restful sleep, which may be a risk factor for cognitive impairment and depression, affecting functionality and quality of life. Insomnia is a very complex multifactorial problem that requires a multi- and interdisciplinary approach. However, it is frequently not diagnosed in older community-dwelling people, increasing the risk of psychological, cognitive, and quality of life alterations. The aim was to detect insomnia and its relationship with cognitive impairment, depression, and quality of life in older community-dwelling Mexicans. An analytical cross-sectional study was carried out in 107 older adults from Mexico City. The following screening instruments were applied: Athens Insomnia Scale, Mini-Mental State Examination, Geriatric Depression Scale, WHO Quality of Life Questionnaire WHOQoL-Bref, Pittsburgh Sleep Quality Inventory. The frequency of insomnia detected was 57% and its relationship with cognitive impairment, depression, and low quality of life was 31% (OR = 2.5, 95% CI, 1.1–6.6. *p* < 0.05), 41% (OR = 7.3, 95% CI, 2.3–22.9, *p* < 0.001), and 59% (OR = 2.5, 95% CI, 1.1–5.4, *p* < 0.05), respectively. Our findings suggest that insomnia is a frequent clinical disorder that is not diagnosed and a significant risk factor for cognitive decline, depression, and poor quality of life.

## 1. Introduction

Sleep disorders are considered a public health problem in the world and are a frequent cause of morbidity and mortality [1]. In this regard, insomnia is the most common sleep disorder in the older population, and constitutes a risk factor for cardiovascular diseases, headaches, cognitive impairment, and depression, among others [2,3,4]. Some studies have reported that insomnia decreases quality of life and interferes with relationships and, if not diagnosed and treated in time, is a risk factor for triggering depression. In this sense, it has been observed that insomnia is strongly associated with other mental and somatic health problems, and with a higher mortality rate [5,6]. The etiology of insomnia is of multifactorial origin, including difficulty in adapting to new changes (retirement, loss of relatives), diseases, psychological distress, polypharmacy, and poor sleep hygiene, among others [2]. In this sense, for the clinical diagnosis of chronic insomnia disorder, the following criteria must be met: (i) symptoms must be present at least three times a week, (ii) for at least three months, and (iii) be associated with daytime consequences. In addition, it is characterized by difficulty to initiate, maintain, or experience refreshing sleep, which increases daily fatigue, negative mood, and poor concentration. The accepted sufficient and restorative sleep time in old age is usually 6 to 7.5 h [7].

Three important factors involved in the symptoms of insomnia have been pointed out: (i) difficulty initiating or maintaining sleep, waking up very early in the morning; (ii) poor sleep quality, and (iii) daytime sleepiness. These factors alter social, family, and work life, causing fatigue, memory, concentration, and attention failures, poor school or occupational performance, mood disorders, or irritability [8].

The prevalence of insomnia is highly variable and depends on the diagnostic criteria used. In this sense, the magnitude reported in adult subjects is very inconsistent, ranging from 5% to 57.7% [9]. On the other hand, aging per se is a risk factor for insomnia; hence, the prevalence is significantly higher in older adults; however, very contrasting magnitudes relative to geographic region have also been reported. In this regard, in older adults from Taiwan, Su et al. (2004) detected a prevalence of only 6% in 2045 noninstitutionalized older individuals aged 65 years or above of an urban community from Taiwan [10], in contrast Tsou (2013), who found 41% (women, 63.3% and men, 36.7) of 1358 Taiwanese individuals (aged ≥ 65 years) [11]. Likewise, El-Gilany et al. (2017) reported a prevalence of 62.1% in a cross-sectional study carried out in 1059 older adults from Egypt [12]. In Mexico, several authors have reported a prevalence that fluctuates between 40 and 62% [13,14,15]. In this context, it is evident that insomnia is not an alteration inherent to aging, since sociocultural, environmental, lifestyle, mental health, and state of health factors are determinants [16].

Insomnia has physical, psychological, cognitive, and social repercussions. In this sense, several studies have found that insomnia is a risk factor for cognitive impairment and even dementia; Cricco et al. (2001) reported a 49% risk for cognitive impairment in a cohort of older adults with chronic insomnia from the USA (OR = 1.49, 95% CI 1.03–2.14) [17]; likewise, Resciniti et al. (2021) found that for each one-unit increase in the insomnia symptom index, there was a five percent greater hazard of mild cognitive impairment (HR = 1.05; 95% CI: 1.04–1.06) and dementia (HR = 1.05; 95% CI: 1.03–1.05) [18]. Insomnia has also been linked to depression and decreased quality of life and daily functioning [19].

Although there is sufficient scientific evidence on the relevance of sleep for physical, psychological, and social health at all stages of life, including old age, insomnia is often a clinical disorder that is underdiagnosed by the general practitioner, assuming that it is a normal change related to aging [20,21,22].

Therefore, the aim of this study is to detect insomnia and its relationship with cognitive impairment, depression, and quality of life in older Mexican adults residing in the community.

## 2. Materials and Methods

### 2.1. Subjects and Design

With prior informed consent, an analytical and comparative study was carried out in a sample of 107 older adults ≥ 60 years old from Mexico City, without comorbidities, not having dementia, without medical and/or psychological treatment for insomnia. The Committee of the Universidad Nacional Autónoma de México (UNAM) Zaragoza Campus approved the research protocol for this study (PAPIIT IN-308620).

### 2.2. Insomnia Measurement

The detection of insomnia was carried out through the application of the Athens Insomnia Scale (AIS). This is a questionnaire structured by 8 Likert-type questions with 4 response options, each with a value of 0–3. It has a minimum overall score of 0 and a maximum of 24; the cut-off points are: 0–7 points is normal and 8 points or more means the presence of insomnia [23].

### 2.3. Sleep Quality Measurement

The Pittsburg Sleep Quality Index (PSQI) was applied. The questionnaire aims to determine the quality of sleep through the assessment of 7 components (subjective sleep quality, sleep latency, sleep duration, habitual sleep efficiency, sleep disturbances, use of sleeping medications, daytime dysfunction). The instrument is divided into two parts: the first consists of 4 questions that evaluate objective sleep data (time taken to fall asleep, time taken to wake up, sleep latency, and hours of sleep); the second evaluates subjective aspects, consists of 14 Likert-type questions with 4 response options that have a value of 0–3 points. The questions corresponding to each of the 7 components are scored and then a sum of their scores is made to obtain the overall rating of the index. The instrument has a minimum score of 0 and a maximum of 21 points; the cutoff point is 0–4 points: good quality of sleep, and 5 points or more indicates poor sleep quality [24].

### 2.4. Cognitive Function Measurement

The Mini-Mental State Examination (MMSE) was applied as a neuropsychological screening test. It is structured by 30 questions that assess 5 areas: orientation, registration, attention and calculation, language, and delayed memory. Every question answered correctly has a value of 1 and incorrect answers score 0. It has a minimum score of 0 and a maximum score of 30 points, where 24 points or more is normal cognitive functioning, and 0–23 points means cognitive impairment [25].

### 2.5. Depression Measurement

The Geriatric Depression Scale (GDS) was applied as a scale for measuring probable depression according to the DSM-IV-TR criteria and is made up of 30 items, the response options are dichotomous, and the score is 0–30. The result is considered probable depression from 11 ≥ points [26].

### 2.6. Quality Life Measurement

The Quality of life Questionnaire (Spanish version) was applied. It is made up of 26 questions, 24 of which are grouped into 4 domains: physical health, psychological, social relations, and environment. The first 2 items refer to the overall quality of life and the person’s perception of health. The response scale is Likert-type, with 5 options. The total score for each domain is 0–100. Questions 3, 4, and 26 are scored in reverse order. The global score is 130 points, which is weighted on a scale of 100 [27]. The instrument is rated to classify poor, average, and good quality of life considering the crude score by area and overall [28].

### 2.7. Satisfaction with Life Scale

The Satisfaction with Life Scale (SWLS) was adapted for the Mexican population. The items on the scale include the following: (i) For most things, my life is close to my ideal; (ii) The conditions of my life are excellent; (iii) I am satisfied with my life; (iv) So far, I have got the things that are important to me in life; and (v) If I were born again, I would change almost nothing in my life. The possible responses were one of three choices: 1 = agree, 2 = neither agree nor disagree, and 3 = disagree. Each item is scored from 1 to 3, so the total score is 15 [29].

### 2.8. Statistical Analysis

Data were analyzed using descriptive statistics, the mean and standard deviation (SD), frequencies (f), and percentages (%). We performed a chi-square comparison test and t student test. A *p* value of <0.05 was considered statistically significant. Moreover, a risk estimator odds ratio (OR) with 95% confidence interval was performed; *p* values were determined using SPSS version 16.0 (IBM, Armonk, NY, USA).

## 3. Results

Table 1 presents the data from the analysis of descriptive measures of the total scores of each of the instruments used to detect insomnia, cognitive function, depression, quality of life, and satisfaction with life, as well as the prevalence of insomnia, poor sleep quality, mild cognitive impairment, depression, low quality of life, and low satisfaction with life.

Of the 61 people with insomnia detected using the AIS, 52 were women and 19 men; 36 single or widowed people and 25 married. Likewise, 21 reported arterial hypertension, 14 hypertension and diabetes, 6 diabetes, and 20 were healthy. Regarding educational level, 25 had a low level (1 to 6 years), 13 medium (7 to 9 years) and 23 high (10 to 12 years) (Table 2).

Regarding the relationship of insomnia detected using the “Athens Insomnia Scale”, it was observed that 31% (19/61) of the people also had mild cognitive impairment (MCI) (OR = 2.5, 95% CI, 1.6–6.0); likewise, 41% (25/61) were identified as struggling with depression (OR = 7.3, 95% CI, 2.3–22.9, *p* < 0.001); 59% (36/61) (OR = 2.5, 95% CI, 1.1–5.4, *p* < 0.05) with low quality of life; and 28% (17/61) (OR = 3.2, 95% CI, 1.1–9.4, *p* < 0.05) with low life satisfaction. In this sense, in the women detected with insomnia, an MCI frequency of 33% (17/52) was observed (OR = 3.9, 95% CI, 1.2–12.7, *p* < 0.05); in addition, 44% (23/52) (OR = 8.7, 95% CI, 2.4–32.0, *p* < 0.001) had depression; 60% (31/52) (OR = 2.3, 95% CI, 1.1–5.5, *p* < 0.05) low quality of life; and 27% (14/52) (OR = 2.9, 95% CI, 1.0–9.9, *p* < 0.05) low satisfaction with life. Regarding age (≥75 years), 50% (11/22) of those detected with depression had MCI (OR = 5.3, 95% CI, 1.2–23.7, *p* < 0.05) and 36% (8/22) (OR = 10.3, 95% CI, 1.1–92, *p* < 0.05) depression. Likewise, regarding marital status (single or widowed), 39% (14/36) of those detected with insomnia had MCI (OR = 4.9, 95% CI, 1.2–19.3, *p* < 0.05); 39% (14/36) depression (OR = 4.9, 95% CI, 1.2–19.3, *p* < 0.05); 67% (24/36) low quality of life (OR = 3.8, 95% CI, 1.3–10.9, *p* < 0.05); and 31% (11/36) (OR = 5.3, 95% CI, 1.1–26, *p* < 0.05) low life satisfaction. Of the people detected with insomnia with some disease (diabetes mellitus or arterial hypertension), only 43% (15/35) (OR = 13.5, 95% CI, 1.6–112, *p* < 0.01) were observed as having depression. Similarly, 52% (13/25) of people with insomnia and a low educational level had depression (OR = 7.6, 95% CI, 1.4–40.5, *p* < 0.01) (Table 3).

On the other hand, no statistically significant association was found between sleep quality and MCI when using the “Pittsburg Sleep Quality Index”; however, 32% (26/81) of people with poor sleep quality were observed as having depression (OR = 3.6, 95% CI, 1.1–13.2, *p* < 0.05); 62% (50/81) (OR = 12.4, 95% CI, 3.4–44.6, *p* < 0.001) low quality of life; and 27% (22/81) (OR = 1.4, 95% CI,1.2–1.6, *p* < 0.01) low life satisfaction. Regarding sex (women), a statistical relationship was found with depression (OR = 5.3, 95% CI, 1.1–24.7, *p* < 0.05); low quality of life (OR = 10.1, 95% CI, 2.7–37.7, *p* < 0.001); and low life satisfaction (OR = 1.4, 95% CI, 1.2–1.6, *p* < 0.01). However, age (≥75 years), marital status, comorbidities, and educational level (low) only found a statistically significant relationship with low quality of life and low life satisfaction (Table 4).

When evaluating the relationship insomnia has with the scores of cognitive impairment, depression, quality of and satisfaction with life, and quality of sleep, lower scores were observed in older adults with insomnia compared to those without, being statistically significant with a *p* < 0.05 (Table 5).

**Figure 1:**
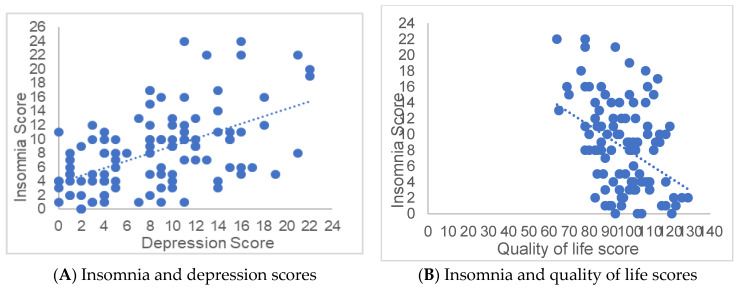
The graphs show the statistically significant positive correlation between the scores of the insomnia scales with that of depression (r = 0.53, *p* < 0.01) (**A**), together with a statistically significant negative correlation between the insomnia score with that of quality of life (r = −0.39, *p* < 0.05) (**B**).

Likewise, a statistically significant positive correlation was observed between the scores of the insomnia and depression scales (r = 0.53, *p* < 0.01), coupled with a negative correlation between insomnia and quality of life (r = −0.39, *p* < 0.05) (Figure 1).

**Figure 2:**
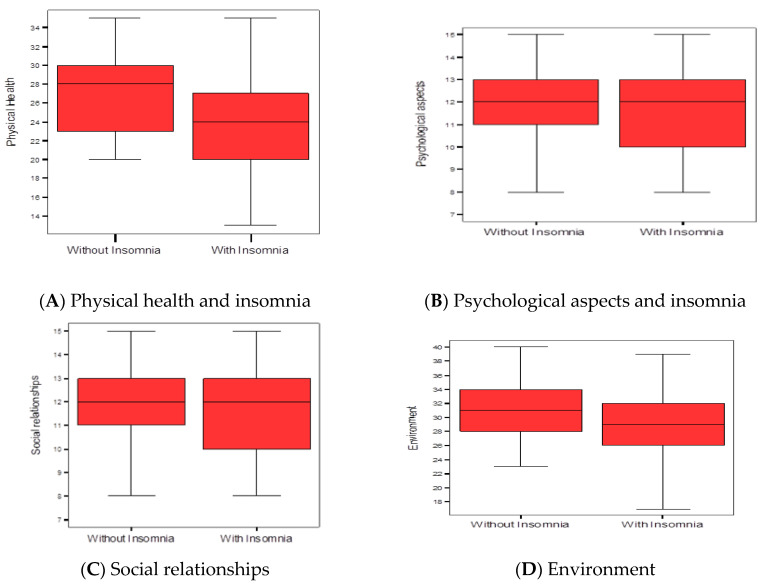
Relationship between insomnia using dimensions of the quality of life scale. (**A**) Physical health, score without insomnia 27.2 ± 3.9 vs. with insomnia 24.0 ± 5.2, *p* < 0.001. (**B**) Psychological aspects, score without insomnia 23.4 ± 3.0 vs. with insomnia 21.1 ± 4.6, *p* < 0.01. (**C**) Social relationships, score without insomnia 12.1 ± 1.9 vs. with insomnia 12.0 ± 1.9, *p* > 0.05. (**D**) Environment, score without insomnia 31.0 ± 4.4 vs. with insomnia 29.0 ± 4.8, *p* < 0.05. Student’s *t*-tests.

Regarding the average values of the different dimensions of quality of life and the relationship with insomnia, it was detected that the group with insomnia obtained lower values in the physical health and psychological aspects and environment dimensions, showing statistically significant differences between both groups (*p* < 0.05) (Figure 2).

## 4. Discussion

The clinical diagnosis of insomnia is framed within the criteria established in the “*Diagnostic and Statistical Manual of Mental Disorders, DSM-5*”: “(i) Dissatisfaction with sleep quantity or quality, associated with one or more of the following: (a) difficulty initiating sleep; (b) difficulty maintaining sleep—characterized by frequent awakenings or problems returning to sleep after awakenings; (c) early morning awakening with inability to return to sleep; (ii) Clinically significant distress or functional impairment; (iii) Occurs at least three nights per week and be present for at least 3 months, despite adequate opportunity for sleep; (iv) Cannot be attributable to the physiologic effects of a substance nor be explained predominantly by a coexisting psychiatric or medical illness” [30]. However, it is frequently underdiagnosed in older adults, especially because it is considered a disorder inherent to aging, which is why general practitioners frequently do not detect this disorder, since some older adults compensate for it and take afternoon naps [2]. For this reason, the need to detect this alteration in the undiagnosed population has been proposed, in order to provide timely and effective treatment and avoid the repercussions of insomnia on cognitive deterioration, depression, and quality of life, among others. In this sense, screening instruments are an alternative for detection [31]; we use the “Athens Insomnia Scale” for the detection of insomnia, complemented with the “Pittsburg Sleep Quality Index” to strengthen the reliability of diagnostic screening.

Insomnia is one of the most prevalent sleep disorders in older adults. However, the prevalence is highly variable, even in populations from the same country. In this sense, there are contrasts, for example, the 6% prevalence of insomnia in older adults in Taiwan observed by Su et al. (2004) [10] and 41% found by Tsou (2013) [11], also in the Taiwanese population of older adults [11]. Therefore, it is evident that insomnia is a very complex multifactorial health problem that requires timely diagnosis and treatment.

In our study, a prevalence of insomnia of 57% was detected, which was similar to that reported by Moreno-Tamayo et al. (2021), who found a 63.9% prevalence of insomnia (<7 h) with an average of 6.04 (±1.5) hours of sleep per night in older adults in Mexico City [15]. In contrast, results of the 2016 Mexican National Halfway Health and Nutrition Survey reported that 28 to 30% of people over 60 years of age sleep ≤ 6 h [32], although the difference may be due to the cut-off point for diagnosis, the highest frequency of insomnia in Mexico City compared to that reported at the national level may be due to the environmental stress to which older adults living in Mexico City are exposed. Environment and culture significantly influence the frequency of insomnia. In this regard, the highest frequencies of insomnia have been reported in Brazil (79.8%), followed by South Africa (45.3%), Eastern Europe (32%), Asia (28.3%), and Western Europe (23.2%). Likewise, sex (women), age (older adults), place of residence (urban), shift workers (at night), and patients with coexisting medical complaints are the main risk factors for insomnia [9,33]. In this sense, Manjavong et al. (2017) reported 60% insomnia in subjects from urban residence, middle class in the preretirement age of 50 years, or older adults who worked for Khon Kaen University (KKU), Khon Kaen, Thailand, and their elderly relatives [34]. Another study by Dangol et al. (2020), in older adults in Nepal, observed a frequency of 71% [35] (Supplementary Materials S1), which is higher than was detected in our study, supporting the proposal that the sociocultural context is a risk factor. In addition, the frequency of insomnia is higher in older people due to multiple factors, such as ageism and “self-ageism”, difficulty in adapting to physical changes related to aging, retirement, loss of family members, chronic diseases, and decreases in social support networks and polypharmacies, among others.

Cognitive impairment has been linked with insomnia, although the results in different studies are inconsistent and controversial [36,37,38,39]. In this sense, it has been pointed out that mild cognitive impairment affects approximately 20% of older adults, and many experience insomnia [40]. Moreover, changes in cognitive performance associated with characteristics in sleep have been observed [41]. In addition, female gender, widowhood, benzodiazepine use, and physical pain were significantly associated with insomnia symptoms and the likelihood of cognitive decline [42,43]. In this regard, Lai et al. (2022) found that 31% of older adults with insomnia presented cognitive impairment; moreover, they observed that sleep quality might predict the development of neurocognitive disorders [44]. On the other hand, in a study from the cohort database of the Health and Retirement Study of older adults in the United States conducted by the University of Michigan, Resciniti et al. (2021) found that for each one-unit increase in the insomnia symptom index, there was a five percent greater hazard of MCI (HR = 1.05; 95% CI: 1.04–1.06) and dementia (HR = 1.05; 95 % CI: 1.03–1.05) [18]. Likewise, in a systematic review and meta-analysis carried out on 48 studies (*n* = 4539 total participants) by Wardle-Pinkston et al. (2019), it was found that insomnia was associated with poorer overall cognitive performance (Hedge’s g = −0.24, *p* < 0.001) [45]. It has also been shown that insomnia causes a significant increase in the production of inflammatory cytokines, promoting a chronic inflammatory process, coupled with a decrease in immune function, which increases the risk of acute and chronic noncommunicable diseases, including Alzheimer’s disease [46,47].

In this study, we found that people detected with insomnia have a 1.5-times higher risk (OR = 2.5, CI 95%, 1.1–6.6, *p* < 0.05) to present cognitive impairment, however, we must consider that sex (women), age (75 ≥ years), and marital status (single or widowed) are also risk factors for cognitive decline in old age.

In this context, in a systematic review carried out by Jin et al. (2021), it was found that cognitive behavioral therapy (CBT) is associated with improvements in mild cognitive impairment and dementia. CBT also showed a reduction in insomnia and improvements in sleep quality [48].

In the other hand, insomnia is a significant risk factor for depression. In this sense, in a meta-analysis carried out by Li et al. (2016), which included thirty-four cohort studies involving 172,077 participants with an average follow-up period of 60.4 months, that a relative risk (RR) of 2.7 (95% CI: 1.89–2.71) was found. Likewise, 12 of the 34 studies were carried out in people ≥ 60 years old, observing an RR = 1.87 (95% CI: 1.47, 2.37) [49]. In contrast, in our study, we found an OR = 7.3 (95% CI: 2.3, 22.9) suggesting that people with insomnia have a sixfold increased risk of depression compared to those without. However, it is important to point out that we observed a prevalence of depression of 57%, and sex (women), age (75 ≥ years), marital status, (single or widowed), comorbidities (DM2 and/or AHT), and educational level (low) were also found as risk factors for depression. In contrast, Tsaras et al. (2021), reported 39% of depression in a community of older adults in Greece [50], this difference supports the proposal that although aging per se is a risk factor for insomnia, the environment is determinant. Additionally, depression increases in old age and predisposes to chronic diseases, accelerated brain aging, advanced epigenetic aging, and dementia disorders [51,52].

In this context, although our study is cross-sectional, this wide difference suggests that the sociocultural conditions of our population and the environment significantly increase the risk of depression in older adults with insomnia.

When evaluating the relationship of insomnia with quality of life and satisfaction with life, it was observed that 59% of older adults with insomnia perceive their quality of life as low, and 28% report low satisfaction with life, data that were statistically significant (*p* < 0.05). These results are similar to what it has been observed in other studies; they have found that older adults with symptoms of insomnia and depressive symptoms were at up to 25.8 times greater risk of having a lower quality of life related with health [53,54]. In this sense, a relationship has been observed between stress and insomnia symptoms that negatively affects quality of life in older adults [55]. In a study carried out in the United States, it was found that participants with poor sleep quality and severe insomnia revealed negative effects on quality of life, including reduced functional capacity and increased stress, anxiety, and social isolation [56].

The aforementioned repercussions of insomnia show that sufficient and restful sleep is a fundamental and vital biological function, through which other physiological functions necessary to maintain correct physical and mental homeostasis in the organism are carried out, such as the restitution of cellular energy, tissue renewal, thermoregulation, immune regulation, maintenance of synaptic networks, memory consolidation, and emotion regulation [19,57]. During deep, restful sleep, metabolic demand has been shown to decrease due to decreased oxygen consumption, heart rate, respiratory rate, and blood pressure, muscle tone, and temperature [58,59]. Likewise, it has been reported that memory encoding processes, memory consolidation and reconsolidation, and brain plasticity occur during sleep [60,61].

In this context, it is important to highlight the importance of timely diagnosis of insomnia in family general medicine consultation, since it is frequently underdiagnosed, being erroneously considered a physiological change related to aging and/or justifying it by the nap reported by older adults, without considering that the habit of afternoon naps could be a consequence of insomnia, leading us to a vicious circle [62,63]. In this regard, Gordon et al. (2022) observed a 30% prevalence of insomnia in older adults, of which only 10% reported having discussed sleep with a health professional [64]. In this sense, Ogeil et al. (2020), point out that the main barriers to the diagnosis of insomnia in family medicine practice are the lack of knowledge of physicians to make an accurate diagnosis, and the beliefs of patients who assign little importance to the consequences of insomnia [65].

Regarding treatment for controlling insomnia, the use of anxiolytics and/or hypnotics have been proposed; however, sleep hygiene and healthy lifestyle habits such as physical exercise, diet, leisure, and an active social life are safe and effective alternatives. The efficacy of cognitive behavioral therapy has also been demonstrated [66].

Regarding the main limitations of this study, it can be noted that the sample size was small, so it was not possible to perform a more robust statistical analysis. Nor can the results be generalized, since the sample was at convenience. Likewise, the study is cross-sectional, and the causal direction cannot be asserted, it can only suggest the relationship of insomnia as a risk factor for affective alterations, cognitive impairment, and quality of life. In this sense, we were only able to calculate OR as an estimator of the RR. Therefore, it is necessary to carry out cohort studies with representative samples of rural and urban populations from all over the country to identify the influence of the environment and the sociocultural characteristics of the different population groups on the frequency and determinant factors of insomnia and its relationship with depressive symptoms, cognitive impairment, and quality of life in older Mexican adults.

## 5. Conclusions

Our findings suggest that insomnia is present in one of every two older adults in Mexico City and the majority is not diagnosed, and therefore not receiving treatment. Likewise, this sleep disturbance is a significant risk factor for cognitive decline and depression in older adults. Additionally, it negatively affects perceptions of life quality and satisfaction with life.

## Figures and Tables

**Table 1:** Scores measuring sleep, cognitive function, depression, and quality of life.

	Mean SD	Highest Percentile	Lowest Percentile	Kurtosis	Skew
AIS	8.4 ± 5.6	22	0	−0.57	0.41
PSQI	8.4 ± 4.4	18	1	−0.81	0.39
MMSE	25.8 ± 2.9	30	17	−0.22	−0.56
GDS-30	8.2 ± 5.6	24	0	0.49	0.86
WHOQOL-BREF	96.8 ± 13.7	129	64	−0.35	0.042
SWLS	12.4 ± 2.6	15	5	0.34	−1.0
	Prevalence (%)	Cutoff point			
Insomnia	61/107 (57)	≥8 score			
Poor Sleep Quality	81/107 (76)	≥5 score			
MCI	26/107 (24)	≤23 score			
Depression	29/107 (27)	≥11 score			
Low Quality of Life	53/107 (49)	≤60 score			
Low SWLS	22/107(21)	≤10 score			

Abbreviations: AIS: Athens Insomnia Scale; PSQI: Pittsburg Sleep Quality Index; MMSE: Mini-Mental State Examination; MCI: mild cognitive impairment; GDS-30: Geriatric Depression Scale; WHOQOL-BREF: World Health Organization Quality of Life Short Version; SWLS: Satisfaction with Life Scale.

**Table 2:** Prevalence of insomnia by sex, marital status, comorbidities, and education level.

Variable	With Insomnia Prevalence (95% CI)	OR (95% CI)	*p* Value
Sex			
Women	52/88 59% (0.49–0.7)	1.6 (0.6–4.3)	
Male	9/19 47% (0.23–0.72)	1.0	0.35
Marital status			
Married	25/45 56% (0.4–0.71)	1.0	
Single or widowed	36/62 58% (0.45–0.71)	1.1 (0.5–2.4)	0.79
Comorbidities			
Diabetes	6/17 35% (0.1–0.61)	0.4 (0.1–1.4)	0.17
Arterial hypertension	21/34 62% (0.45–0.79)	1.3 (0.5–3.4)	0.6
Diabetes and hypertension	14/20 70% (0.48–0.92)	1.9 (0.6–5.9)	0.3
Healthy	20/36 56% (0.39–0.73)	1.0	
Educational level			
Low (1–6 years)	25/41 61% (0.45–0.77)	1.3 (0.54–3.0)	0.57
Medium (7–9 years)	13/24 54% (0.36–0.76)	0.9 (0.36–2.7)	0.96
High (10–12 years)	23/42 55% (0.39–0.7)	1.0	

**Table 3:** Relationship between insomnia and MCI, depression, quality of and satisfaction with life.

Variable	MMSE	OR (CI 95%)	GDS-30	OR (CI 95%)	WHOQOL	OR (CI95%)	SWLS	OR (CI 95%)
Insomnia (AIS)								
With insomnia	19/61		25/61		36/61		17/61	
	(31%)	2.5 (1.1–6.6)	(41%)	7.3 (2.3–22.9)	(59%)	2.5 (1.1–5.4)	(28%)	3.2 (1.1–9.4)
Without insomnia	7/46	*p* < 0.05 *	4/46	*p* < 0.001 ***	17/46	*p* < 0.05 *	5/46	*p* < 0.05 *
	(15%)		(9%)		(37%)		(11%)	
Sex (women)								
With insomnia	17/52		23/52		31/52		14/52	
	(33%)	3.9 (1.2–12.7)	(44%)	8.7 (2.4–32)	(60%)	2.3 (1.0–5.5)	(27%)	2.9 (0.9–9.9)
Without insomnia	4/36	*p* < 0.05 *	3/36	*p* < 0.001 ***	14/36	*p* < 0.05 *	4/36	*p* > 0.05
	(11%)		(8%)		(39%)		(11%)	
Age (75 ≥ years)								
With insomnia	11/22		8/22		14/22		7/22	
	(50%)	5.3 (1.2–23.7)	(36%)	10.3 (1.1–92)	(64%)	2.4 (0.7–8.5)	(32%)	3.9 (0.7–22)
Without insomnia	3/19	*p* < 0.05 *	1/19	*p* < 0.05 *	8/19	*p* > 0.05	2/19	*p* > 0.05
	(16%)		(5%)		(42%)		(11%)	
Marital status (single or widowed)								
With insomnia	14/36		14/36		24/36		11/36	
	(39%)	4.9 (1.2–19.3)	(39%)	4.9 (1.2–19.3)	(67%)	3.8 (1.3–10.9)	(31%)	5.3 (1.1–26)
Without insomnia	3/26	*p* < 0.05 *	3/29	*p* < 0.05 *	9/26	*p* < 0.05 *	2/26	*p* < 0.05 *
	(12%)		(12%)		(35%)		(8%)	
Comorbidities (DM2 and AHT)								
With insomnia	11/35		15/35		19/35		9/35	
	(31%)	1.7 (0.5–6.4)	(43%)	13.5 (1.6–112)	(54%)	2.6 (0.8–8.3)	(26%)	6.2 (0.7–53)
Without insomnia	4/19	*p* > 0.05	1/19	*p* < 0.01 **	6/19	*p* > 0.05	1/19	*p* > 0.05
	(21%)		(5%)		(32%)		(5%)	
Educational level (low)								
With insomnia	10/25		13/25		14/25		8/25	
	(40%)	1.5 (0.4–5.5)	(52%)	7.6 (1.4–40.5)	(56%)	1.6 (0.5–5.8)	(32%)	3.3 (0.6–18)
Without insomnia	5/16	*p* > 0.05	2/16	*p* < 0.05 *	7/16	*p* > 0.05	2/16	*p* > 0.05
	(31%)		(13%)		(34%)		(13%)	

Abbreviations: AIS: Athens Insomnia Scale; PSQI: Pittsburg Sleep Quality Index; MMSE: Mini-Mental State Examination; MCI: mild cognitive impairment; GDS-30: Geriatric Depression Scale; WHOQOL-BREF: World Health Organization Quality of Life Short Version; SWLS: Satisfaction with Life Scale. DM2: type 2 diabetes mellitus; AHT: arterial hypertension. We estimated the odds ratio (OR) of experiencing insomnia in individuals with and without mild cognitive impairment, depression, low quality of life, and low satisfaction with life; *p* < 0.05 *; *p* < 0.01 **; *p* < 0.001 ***.

**Table 4:** Relationship between sleep quality and MCI, depression, quality of and satisfaction with life.

Variable	MME	OR (CI 95%)	GDS-30	OR (CI 95%	WHOQOL	OR (CI 95%)	SWLS	OR (CI 95%)
Sleep Quality (PSQI)								
Poor = 81	20/81		26/81		50/81		22/81	
	(25%)	1.1 (0.4–3.1)	(32%)	3.6 (1–13.2)	(62%)	12.4 (3.4–44.6)	(27%)	1.4 (1.2–1.6)
Good = 26	6/26	*p* > 0.05	3/26	*p* < 0.05 *	3/26	*p* < 0.001 ***	0/26	*p* < 0.01 **
	(23%)		(12%)		(12%)		(0%)	
Sex (women)								
With poor sleep quality	16/67		24/67		42/67		18/67	
	(24%)	1.0 (0.3–3.2)	(36%)	5.3 (1.1–24.7)	(63%)	10.1 (2.7–37.7)	(27%)	1.4 (1.2–1.6)
With good sleep quality	5/21	*p* > 0.05	2/21	*p* < 0.05 *	3/21	*p* < 0.001 ***	0/21	*p* < 0.01 **
	(24%)		(10%)		(14%)		(0%)	
Age (≥75 years)								
With poor sleep quality	10/29		6/29		20/29		9/29	
	(26%)	1.1 (0.25–4.4)	(21%)	0.9 (0.65–1.4)	(69%)	11.1 (2.0–61.0)	(31%)	1.5 (1.1–1.9)
With good sleep quality	4/12	*p* > 0.05	3/12	*p* > 0.05	2/12	*p* < 0.01 **	0/12	*p* < 0.05 *
	(33%)		(25%)		(17%)		(0%)	
Marital status (single or widowed)								
With poor sleep quality	12/47		15/47		31/47		13/47	
	(26%)	0.7 (0.2–2.4)	(32%)	3.0 (0.6–15.2)	(66%)	12.6 (2.5–62.7)	(28%)	1.4 (1.2–1.7)
With good sleep quality	5/15	*p* > 0.05	2/15	*p* > 0.05	2/15	*p* < 0.001 ***	0/15	*p* < 0.05 *
	(33%)		(13%)		(13%)		(0%)	
Comorbidities (DM2 and AHT)								
With poor sleep quality	12/40		14/40		24/40		10/40	
	(30%)	1.6 (0.4–6.7)	(35%)	3.2 (0.6–16.5)	(60%)	19.5 (2.3–164)	(25%)	1.3 (1.1–1.6)
With good sleep quality	3/14	*p* > 0.05	2/14	*p* > 0.05	1/14	*p* < 0.01 **	0/14	*p* < 0.05 *
	(21%)		(14%)		(7%)		(0%)	
Educational level (low)								
With poor sleep quality	11/32		13/32		19/32		10/32	
	(34%)	0.7 (0.15–2.9)	(41%)	2.4 (0.4–13.4)	(59%)	5.1 (1.0–28.6)	(31%)	1.5 (1.2–1.8)
With good sleep quality	4/9	*p* > 0.05	2/9	*p* > 0.05	2/9	*p* < 0.05 *	0/9	*p* < 0.05 *
	(44%)		(22%)		(22%)		(0%)	

Abbreviations: AIS: Athens Insomnia Scale; PSQI: Pittsburg Sleep Quality Index; MMSE: Mini-Mental State Examination; MCI: mild cognitive impairment; GDS-30: Geriatric Depression Scale; WHOQOL-BREF: World Health Organization Quality of Life Short Version; SWLS: Satisfaction with Life Scale. DM2: type 2 diabetes mellitus; AHT: arterial hypertension. We estimated the odds ratio (OR) of experiencing insomnia in individuals with and without mild cognitive impairment, depression, low quality of life, and low satisfaction with life; *p* < 0.05 *; *p* < 0.01 **; *p* < 0.001 ***.

**Table 5:** Cognitive function, depression, quality of life, satisfaction with life, and quality of sleep scores by subjects without and with insomnia.

Variable	Without Insomnia	With Insomnia	*p*-Value
	*n* = 46	*n* = 61	
Cognitive function score	26.5 ± 6.7	25.3 ± 2.9	<0.05 *
Depression score	5.0 ± 3.5	10.7 ± 5.6	<0.001 **
Quality of life score	102 ± 12	93 ± 14	<0.001 **
Satisfaction with life score	13.4 ± 1.8	11.7 ± 2.8	<0.001 **
Sleep quality score	5.1 ± 2.3	10.9 ± 3.8	<0.001 **

Student’s *t*-tests *p* < 0.05 *, *p* < 0.001 **.

## Data Availability

The data presented in this study are available on request from the corresponding author.

## Supplementary Materials

The following supporting information can be downloaded at: https://www.mdpi.com/article/10.3390/diagnostics13111889/s1, Supplementary Materials S1: Prevalence of insomnia in different countries by diagnostic criteria or screening instruments. References [67,68,69,70,71] are cited in the Supplementary Materials.
