# Supplementary material for: Detection of Insomnia and Its Relationship with Cognitive Impairment, Depression, and Quality of Life in Older Community-Dwelling Mexicans
Source: Diagnostics (Basel). 2023 May 28;13(11):1889. doi: 10.3390/diagnostics13111889 (PMC10252556; doi:10.3390/diagnostics13111889)
Supplement: Supplementary file 1 [file diagnostics-13-01889-s001.zip › diagnostics-2252636-supplementary.pdf]

Supplementary Materials S1. Prevalence of insomnia in different countries by diagnostic criteria or screening instruments

**Country (City)**	**Population, age and sex**	**Residence**	**Diagnostic criteria and/or measuring instrument**	**Prevalence**	**Author (year)**
China (Taiwan)	n=2045<br>Age ≥65 years<br>Male n=1156<br>Female n= 889	Community-dwelling in Taipei City	DSM-IV<br>Pittsburgh Sleep Quality Index	Overall 6%<br>Women 8%<br>Men 4.5%	Su et al. (2004) [10]
China (Taiwan)	n=1358<br>Age≥65 years<br>Male n= 601<br>Female n=757	Medical centers in Taipei City	Athens insomnia scale<br>Brief Symptom Rating Scale (BSR-5)	Overal 41%<br>Women 63.3%<br>Men 36.7%	Tsou (2013) [11]
USA (East Boston, New Haven and Iowa)	n=9, 282<br>Age≥65 years<br>Male n= 3,600<br>Female n= 5,682	Established Populations for Epidemiologic Studies of the Elderly (EPESE)	Five questions: (1) have trouble falling asleep, (2) have trouble with waking up during the night, (3) have trouble with waking up too early and not being able to fall asleep again,(4) get so sleepy during the day or evening they have to take a nap and (5) feel really rested when waking up in the morning. (rarely or never, sometimes or most of the time-weighted 0, 1 and 2, respectively)	**East Boston**<br>Overal 37.7%<br>Women 36.4%<br>Men 29.4%<br><br>**New Haven**<br>Overal 27.5%<br>Women 31.1%<br>Men 21.2%<br><br>**Iowa**<br>Overal 23.2%<br>Women 25.4%<br>Men 19.5%	Foley et al. (1995) [67]
USA (Beaver Dam, Wisconsin)	N= 2800<br>Age 53-97 years<br>Mean age 69.3±9.8 years<br>Male n= 1158<br>Female n= 1642	Epidemiology of Hearing Loss Study (EHLS), a population-	Three questions: (1) Have difficulty getting to sleep? (2) Wake up and have a hard time getting back to sleep? (3) Wake up repeatedly?. Participants	Overal<br>Least one insomnia trait<br>49%	Schubert et al. (2002) [68]

		based study of older adults in Beaver Dam, Wisconsin.	could respond never, rarely (1/month), sometimes (2-4 times/month), often (5-15 times/month) or almost always (16-30 times/month). A participant was considered to be positive for an "insomnia trait" if they responded "often" or "almost always." Each participant could therefore have a maximum of three "insomnia traits."	Three.insomnia trais 10%<br><br>Difficulty getting to sleep<br>Women 26.7%<br>Men 13.5%<br><br>Wake up/Hard back to sleep<br>Women 27.8%<br>Men 18.9%<br><br>Wake Repeatedly<br>Women 37.5%<br>Men 34.4%	
Multicenter study<br>Austria<br>Belgium<br>Brazil<br>China<br>Germany<br>Japan<br>Portugal<br>Slovakia<br>South Africa<br>Spain	Overall N= 35,327<br>Mean age 39±15.3<br>Male n= 17,593<br>Female n= 17,734	Urban	Athens Insomnia Scale	Overall 31.6%<br><br>Austria 19%<br>Belgium 36%<br>Brazil 79.8%<br>China 28%<br>Germany 17.4%<br>Japan 28.5%<br>Portugal 21.2%<br>Slovakia 32%<br>South Africa 45.3%<br>Spain 22.4%	Soldatos et al. (2015) [69]
Nepal (Banepa Municipality)	n= 114<br>Age ≥65 years	Urban	Pittsburgh Insomnia Rating Scale (20-item version)	Overall 71.1%	Dangol et al. (2019) [35]

	Male n=55<br>Female n= 59		Minimum score 0 is a good, maximum<br>score of 60 is bad, and score >20 is diagnosed as insomnia		
Egypt (Mansoura District)	N=1,059<br>Age ≥60 years<br>Male n=531<br>Female n=528	Rural	Athens insomnia scale<br>Score of 6 or higher was considered as a positive case for insomnia.	Overall 62.1%<br>Women 70.5%<br>Men 53.9%	El-Gilany et al. (2017) [12]
Multicenter study<br>USA<br>Western Europe (France, Germany, Italy, Spain & UK)<br>Japan	N= 10,132<br>Age ≥ 15 years<br><br>USA n=3,962<br>Male n=2,020<br>Female 1,946<br><br>Western Europe n=5005<br>Male n= 2,402<br>Female n= 2,603<br><br>Japan n=1165<br>Male n=524<br>Female n=641	Urban	Three questions<br>'Have you suffered from sleeping problems in the past 12 months?'<br>Insomnia was defined as having at least one sleep<br>problem (difficulty falling asleep, difficulty staying asleep due to night-time awakenings, waking up early and not being able to go back to sleep, poor quality of<br>sleep) experienced at least several times per week for more than 1 year, with daytime consequences.	Sleep induction problems<br>USA 57%<br>Western Europe 55%<br>Japan 69%<br><br>Sleep maintenance problems<br>USA 78%<br>Western Europe 75%<br>Japan 63%<br><br>Poor sleep quality<br>USA 52%<br>Western Europe 33%<br>Japan 31%	Legér (2008) [70]
Thailand (Khon Kaen)	N=491<br>Age ≥ 50 years	Urban	International Classification of Diseases (ICD10)	Overall 60%<br>Women 60.6%	Manjavong et al. (2016) [34]

	Male n=168<br>Female n=323			Men 58,9%	
Mexico<br>(Nationally representative survey data)	n=8649<br>Age ≥18years	2016 Mexican National Halfway Health and Nutrition Survey	Were asked the following questions (in Spanish) regarding sleep:"In general, how many hours do you sleep daily during the night from Monday to Friday?,";"How do you qualify, in general, the quality of your" In the last 3weeks, have you had difficulty to sleep, to maintain sleep or keep awake sooner than you would like?; "How frequently do you use medications for sleeping (prescribed or over-the-counter)?"	Difficulty sleeping 37%<br>Sleep 6 or less hours 28.5%	Arrona-Palacios & Gradisar (2021) [32]
Multicenter study<br><br>Finland<br>Greece<br>Jordan<br>Lebanon<br>Morocco<br>Mexico<br>Philippines<br>Portugal<br>Sweden<br>Switzerland	Overal N=13, 134<br>Age ≥18years<br><br>Finland n= 1782 (14%)<br>Greece n= 870 (7%)<br>Jordan n= 140 (1%)<br>Lebanon n= 296 (2%)<br>Marocco n= 2476 (19%)<br>Mexico n= 3021 (23%)<br>Philippines n= 1417 (11%)<br>Portugal n= 1727 (13%)<br>Sweden 542 (4%)<br>Switzerland 853 (6%)	Insomnia in primary care physicians'<br><br>EQUINOX (evaluation of daytime quality impairment by nocturnal awakenings in outpatient's experience)	DSM-IV<br>American Academy of Sleep Medicine<br><br>Questionnaire<br>Difficulty initiating sleep (DIS)<br>Difficulty maintaining sleep (DMS)<br>Early morning awakenings (EMA)<br>Nonrestorative sleep (NRS)	Overall<br>Non-medically treated insomnia 42%<br><br>DIS 78%<br>DMS 80.2%<br>EMA 66.9%<br>NRS 78.6%<br><br>Finland 10.05%<br>Greece 7.43%<br>Jordan 1.11%	Léger et al. (2010) [71]

				Lebanon 3.54%<br>Morocco 21.50%<br>Mexico 25.44%<br>Philippines 9.37%<br>Portugal 9.90%<br>Sweden 5.57%<br>Switzerland 6.09%	
Mexico (Mexico City)	N=1678<br>Age ≥60years<br>Male n= 838<br>Female n=840	Community-dwelling in Mexico City<br>Urban	Athens insomnia scale<br>Pittsburgh Sleep Quality Index<br>Epworth scale<br>≤ 6 h 59 min	Overall<br>Insomnia 30.8%<br>Women 33.7%<br>Men 27.8%<br>≤ 6 h 59 min 63.9%	Moreno-Tamayo et al. (2021) [15]
